# Editorial: Translational research for better diagnosis and treatment of endometrial cancer

**DOI:** 10.3389/fonc.2023.1305140

**Published:** 2023-10-19

**Authors:** Andrea Romano, Andrzej Semczuk, Janina Tokarz, Tea Lanišnik Rižner

**Affiliations:** ^1^ Department of Gynaecology, Maastricht University Medical Centre (MUMC), Maastricht, Maastricht, Netherlands; ^2^ GROW – School for Oncology and Reproduction, Maastricht University, Maastricht, Netherlands; ^3^ Department of Gynaecology, Lublin Medical University, Lublin, Poland; ^4^ Helmholtz Zentrum München, German Research Center for Environmental Health, Institute for Diabetes and Cancer, Neuherberg, Germany; ^5^ German Center for Diabetes Research (DZD), Neuherberg, Germany; ^6^ Institute of Biochemistry and Molecular Genetics, Faculty of Medicine, University of Ljubljana, Ljubljana, Slovenia

**Keywords:** endometrial cancer, gynaecology, proteomics, metabolomics, omics, biomarkers

Endometrial cancer (EC) is the most frequent gynaecological malignancy in developed countries and represents a clinical challenge, especially in terms of early diagnosis and risk stratification of patients. Early diagnosis is fundamental to ensure a good prognosis, long survival and good quality of life, whereas an accurate (and ideally) pre-operative stratification of patients based on risk of recurrence is a prerequisite to appropriately decide on the extent of surgery and on the adjuvant care.

Currently, both these aspects are not optimal. An invasive endometrial histology is the gold standard for diagnosis, and there are no valid non-invasive methods; also, patient stratification is based on histopathology and surgical findings. To tackle these limitations and to develop non-invasive diagnostic/prognostic tools, the BioEndoCar project was launched in 2018 (funded by EU framework programme Horizon2020). Six European partners and five collaborating centres joined forces to prospectively collect blood specimens from patients and controls, to perform metabolomics and proteomics analyses in plasma samples in search for minimally invasive diagnostic and prognostic biomarkers, and to model the data for the development of prediction algorithms. The BioEndoCar project concluded with a two-day international symposium that was held in Portorož (Slovenia, March 2022) focussing on state-of-the-art *omics* technologies, biobanking, translational research and clinical management in the context of EC.

This symposium was the birth of the Research Topic for Frontiers on Oncology aiming to transfer the current challenges and discussions on EC into a dedicated collection of articles. The Research Topic focussed on translational research to improve diagnosis/prognosis and treatment of EC. We collected high quality Original Research articles, Systematic Reviews and Narrative Reviews describing different aspects of translational research, with particular emphasis on *omics* profiling and multi-*omics* in tissues and physiological fluids. Over 120 authors from China, Europe (Czech Republic, Germany, Poland, Slovenia, The Netherlands), India, Switzerland, UK and United States contributed to the Topic with 15 papers.

In the present entitled ‘*Translational research for better diagnosis and treatment of endometrial cancer*’, we coherently organised these 15 papers in four sections: 1. Pathogenesis, classification and treatment; 2. Comorbidities; 3. Diagnostic/prognostic models based on histological and blood biomarkers; and 4. Diagnostic/prognostic models based on imaging data.

The first Section (Pathogenesis, classification and treatment) consists of five contributions (one review and four original papers). The review by Zhang et al. summarises the expression, regulation, and functions of glucose transporters (GLUTs) in human EC. The authors review the upstream regulators of GLUTs, and briefly discuss their functions in tumour growth and invasion. The impact of GLUTs in the context of treatments and ongoing clinical trials is also discussed. The authors conclude that GLUTs overexpression may be implicated in insensitiveness to hormone therapy or resistance to chemoradiotherapy.

The original articles focus on intracellular signalling and tumour immune microenvironment. Ledinek et al. analyse the possible interconnection between Wnt signalling and epithelial-to-mesenchymal-transition (EMT) among 64 EC specimens. Markers of Wnt signalling and EMT correlate significantly with hormone receptor status, although no further correlation was found with clinic-pathological features or integrated molecular subgroups. The authors conclude that the correlation between hormone receptors, Wnt signalling and EMT confirms the intimacy between these pathways in EC. Dai et al. explore the immune cell infiltration in tumour samples from a small cohort of EC patients that were classified into four molecular subtypes (according to transPORTEC). The profiles of infiltrating immune cells differed between tumours with distinct molecular subtypes, implying distinct immune reactions (normal responses, absence or suppressed responses), and potentially explaining the differences in prognosis and therapy efficiency among different EC cancer subtypes.

The last two papers describe less-common forms of EC, specifically clear cell and serous carcinoma. In the first study, Cui et al. develop nomograms to predict overall survival (OS) at 3-, 5-, and 10-year after diagnosis using a retrospective cohort of 1778 cases. Age at diagnosis, marital status, stage, tumour size and surgery were independent predictors for OS among women with FIGO stage I/II. Age at diagnosis, stage, lymph node involvement, distant metastasis, tumour size, surgery, radio- and chemo-therapy were all independent OS predictors for FIGO stage III/IV. The authors conclude that the predictive models they built may be valuable tools in clinical practice.

The second study focus on serous carcinoma, an aggressive subtype of endometrial carcinoma. Alessandrino et al. examined associations between genomics and metastatic patterns in 67 patients (including Hispanic and black subjects) and observe lower overall survival in patients with presence or recurrence of metastases to the liver and *AKR1D1A* mutations. This study underscores the importance of genomic studies for individualised treatment of these patients.

Section 2 focuses on comorbidities associated with EC and features one review on the impact of adipose tissue and one original paper on the impact of type 2 diabetes on EC. The systematic review of van den Bosch et al. explores the association between patient characteristics and the distribution of adipose tissue. Eleven retrospective studies are included and indicate that the distribution of adipose tissue (visceral versus subcutaneous) significantly correlates with obesity, cancer histology, metastasis, sex steroid levels and survival. The work by Njoku et al. aims to investigate whether pre-existing diabetes can affect survival outcomes in patients with EC. The authors included over 500 subjects and demonstrated that pre-existing type 2 diabetes confers an increased risk of death among EC patients.

Section 3 features studies (one review and three original contributions) on diagnostic or prognostic models that included histological and/or blood biomarkers. The systematic review by Romano et al. describes the current state-of-the-art in diagnostic and prognostic biomarkers for EC. The review provides a brief description of technological and data analyses aspects, and continues by describing all studies that used proteomics and/or metabolomics for diagnostic and prognostic biomarker discovery. Vinklerová et al. address in their study the problem of preoperative risk stratification. The authors validate a previously developed Bayesian network model for preoperative risk stratification of EC patients (ENDORISK) developed within the ENITEC network (European Network of Individualized Treatment of Endometrial Cancer). In a cohort of 445 patients, ENDORISK, focusing on lymph node metastases and disease-specific survival, has good predictive value for low-risk but underestimates the risk among high-risk patients. This confirms that further improvements of the model are needed by including additional preoperative features (molecular classification, myometrial, cervical invasion, distant metastases, etc.) before its implementation in clinical practice. The original article by Roškar et al. includes 202 subjects (91 cases and 111 controls) and shows that plasma levels of leptin are significantly higher in patients with type 1 EC than in control patients, whereas IL-8 is higher in type 2 ECs versus control patients. The authors further develop a model based on age, IL-8, leptin, and the angiogenic factor G-CSF with good diagnostic accuracy. This section concludes with the study by He et al., who, through mining the TCGA database combined with *in vitro* investigations, explore the relation between KNL1 expression, patient prognosis and the effect on cell proliferation, invasion and metastatic potential. The authors conclude that KNL1 can be a prognostic and diagnostic biomarker in patients with EC.

Section 4 includes one review and three original papers where imaging techniques are used to develop diagnostic or prognostic models. The molecular classification of EC subordinates the histologic subtype to the molecular class. Fremond et al. suggest that Deep Learning (DL) could open a new door to refining the current EC classification by integrating histologic and molecular data. To date two studies have provided proof of principle for the prediction of molecular classes from H/E slide images by DL, albeit with relatively poor performance, that should improve with dataset size and quality and advances in DL technology. Automated DL models could provide a cost-effective alternative, accelerate the diagnostic process and advance treatment. The study by Zhang et al. explores atypical endometrial hyperplasia (AEH), which is considered a direct precursor of EC, with concurrent EC diagnosed in approximately 40% of patients undergoing hysterectomy for AEH. The authors develop and annotate a multimodality MRI-based radiomic-clinical model to noninvasively distinguish EC from AEH. This model includes nulliparity status, endometrial thickness, and a combined radiomicroscopic signature with excellent performance. Further validation of this model in multicentre studies is needed, and the properties of the model can be further improved by combining it with genomic data. The diagnosis of EC relies currently on a combination of pre-operatively collected data such as age, BMI, blood-based tumour markers or imaging results, which are semi-structured or unstructured data. Feng et al. developed a clinical decision support system based on machine learning algorithms that include 16 features to assist physicians in classifying histology, stage, and grade of EC patients. The models showed different performances depending on the algorithm, and have highest accuracy if combined with a physician’s judgement. Precise pre-operative EC tumour grade prediction is essential for risk stratification and treatment. Thus, Yue et al. use multiparametric magnetic resonance imaging (MRI) to determine radiomics features, which are the basis for calculating a radiomics score used to design a nomogram. The nomogram could improve the accuracy of recognizing a high-grade tumour prior to surgery in comparison to dilation and curettage and had a good net benefit according to decision curve analysis.

In conclusion, this e-book presents an up-to-date overview of the current diagnostic and prognostic tools that are under development in translational research and that hopefully will find their way to a clinical applicability in the near future. The editors hope that this e-book and the studies described herein will represent milestones in research and inspiration for all scientists and clinicians working in the field of EC to improve the care of women with this disease in the future.

## Author contributions

AR: Writing – original draft, Writing – review & editing. AS: Writing – original draft, Writing – review & editing. JT: Writing – original draft, Writing – review & editing. TL: Writing – original draft, Writing – review & editing.

